# Universal-offer HIV testing amongst patients undergoing blood tests in primary care: results from a multi-centre study

**DOI:** 10.1093/fampra/cmag038

**Published:** 2026-06-30

**Authors:** Rhys D Wenlock, Sean Perera, Jaime H Vera, Gillian Dean

**Affiliations:** The Lawson Unit, University Hospitals Sussex NHS Foundation Trust, Brighton, BN2 5BE, United Kingdom; The Lawson Unit, University Hospitals Sussex NHS Foundation Trust, Brighton, BN2 5BE, United Kingdom; The Lawson Unit, University Hospitals Sussex NHS Foundation Trust, Brighton, BN2 5BE, United Kingdom; Department of Global Health and Infection, Brighton and Sussex Medical School, University of Sussex, Brighton, BN1 9RH, United Kingdom; The Lawson Unit, University Hospitals Sussex NHS Foundation Trust, Brighton, BN2 5BE, United Kingdom

**Keywords:** HIV, screening, implementation, acceptability, feasibility, primary healthcare

## Abstract

**Background:**

The 2016 NICE guidelines recommend offering HIV testing alongside routine blood tests in areas of high HIV prevalence (>2 per 1000 adults aged 18–59). Despite this, implementation in primary care has been limited.

**Method:**

A universal-offer HIV testing intervention was implemented across seven general practices in Brighton and Hove. Data were collected on all blood test appointments between June 2022 and February 2024, including demographics, whether an HIV test was offered, patient acceptance, and test results. Mixed-effects logistic regression was used to assess factors associated with test offer and uptake. An electronic survey was disseminated to staff to gather views on feasibility and acceptability.

**Results:**

Seven sites were recruited, although only four successfully engaged and provided complete data. A total of 6105 HIV tests were conducted, with one new diagnosis identified. HIV testing was offered to 45% of eligible patients, with 74% accepting. Older adults were significantly less likely to be offered a test [aOR 71–80: 0.79 (95% CI: 0.69–0.91); 81+: 0.50 (0.42–0.60)]. Amongst those offered testing, patients aged over 50 were less likely to accept [aOR: 0.72 (0.57–0.91)], with the lowest uptake amongst those aged 81+ [aOR: 0.44 (0.32–0.61)]. Staff reported no barriers to feasibility or patient acceptability.

**Conclusion:**

Routine HIV testing in primary care is well-accepted and can identify new cases. However, site engagement can be challenging, with closer monitoring, support, and rigorous on-boarding processes required.

Key messages

- We implemented a universal-offer HIV testing strategy alongside routine blood tests in primary care in Brighton and Hove and evaluated its feasibility and acceptability.
- HIV testing was highly acceptable to patients, with 74% accepting a test; however, uptake was lower amongst adults aged over 50 years.
- Of seven participating sites, four completed the study. At these sites, HIV testing was offered to 45% of eligible patients, with older age associated with a lower likelihood of being offered a test, and overall testing rates declining over time.
- One previously undiagnosed HIV infection was identified amongst 6105 tests, corresponding to a number needed to test of 6079 (95% CI: 1091–240 384). Cost-effectiveness is likely to depend on the local prevalence of undiagnosed HIV.
- Staff reported no major barriers to implementation; however, further investigation is needed to understand site withdrawal and fully assess feasibility.

## Introduction

Ending HIV transmission in the UK has been a central public health goal since the publication of the national HIV action plan in 2019 [1]. Central to this ambition is the identification of individuals living with undiagnosed HIV, who contribute to onward transmission disproportionately, due to unsuppressed viral loads [2, 3].

In 2016, the National Institute for Health and Care Excellence (NICE) issued guidelines recommending that in areas with high HIV prevalence, all patients undergoing blood tests in primary care should also be offered a HIV test. However, uptake in primary care has been limited. Barriers include time constraints during consultations, perceived stigma, and a lack of clarity about how to integrate testing into routine workflows [4, 5].

Meanwhile, there is growing evidence that HIV testing outside of traditional sexual health services is both feasible and effective. HIV testing has been routinely offered in antenatal clinics since 1999, where it has been shown to be feasible, acceptable, and effective with over 99% of women screened during pregnancy. Moreover, in 2022, ED opt-out HIV testing was implemented in the UK and has identified a substantial number of people living with undiagnosed HIV [6–8]. This has helped to normalize routine HIV testing in non-specialist settings and demonstrates the potential to test while minimizing disruption.

Despite this, primary care remains an underutilized setting for HIV testing. General practice is often the first point of contact for patients living with undiagnosed HIV and offers a critical opportunity for earlier detection [9]. The RHIVA2 trial previously demonstrated that HIV testing upon registration in primary care increased testing rates and case detection [10, 11]. However, more than a decade on, there has been limited progress and no widespread implementation of routine HIV testing.

To address this gap, we first conducted a 3-week pilot of universal-offer HIV testing for patients undergoing blood tests in a single centre in general practice in Brighton and Hove [12]. Although in only one centre, the pilot identified that after appropriate training healthcare workers were able to offer HIV testing alongside routine blood test appointments, that patients were mostly accepting of them and staff identified no significant barriers. This multi-centre study builds on the initial pilot and primarily aims to assess the feasibility of implementing testing across multiple centres (i.e. whether tests can be reliably offered) and the acceptability of testing to patients (i.e. whether patients accept testing once offered). In addition, we sought to characterize the ability of universal-offer HIV testing to identify people living with HIV, in order to inform future evaluations of cost-effectiveness.

## Methods

### Study design

Between June 2022 and February 2024, we conducted a multi-centre study of universal-offer HIV testing in seven primary care centres in Brighton and Hove. All blood test appointments conducted by each practice were eligible for inclusion regardless of indication for the blood test. Posters outlining the study were on display in the waiting rooms of the centres. We initially planned to recruit three primary care centres (one from pilot and two additional) based upon existing relationships and then expanded to include a further four. In total, nine centres were contacted for potential involvement (two non-reply). All costs associated with the study were covered by the research funding.

All participating healthcare workers underwent training, led by a member of the research team on universal-offer HIV testing. Universal-offer refers to a policy whereby all patients attending for blood tests were offered a HIV test, with phrasing such as ‘All patients now coming in for blood tests are being offered a HIV test, is that ok?’. Adults over the age of 18 were eligible. Patients were to be offered a HIV test once every 12 months. For patients that accepted a HIV test, an additional blood tube was taken and tested by a fourth generation HIV 1&2 antibody/p24 antigen test processed at The Doctors Laboratory [https://www.tdlpathology.com]. Reactive tests were flagged in real time by The Doctors Laboratory to the research team, who passed the patient details on to the sexual health contact tracing team in Brighton and Hove. Clinicians were able to capture the offer and acceptance of a HIV test in an adapted GP blood test template as part of their routine electronic healthcare record (SystemONE or EMIS). This was developed with the healthcare workers at each site, to increase usability, with training and support provided.

In February 2023 due to the lower-than-expected testing numbers, and to encourage new primary care sites to enrol, a decision was made to provide financial incentives to new and existing centres. These were decided by the research team based upon the remaining budget to be allocated and the expected number of tests to be performed. Practice managers and participating healthcare workers were informed of this incentive. These were £2 per HIV test performed; £200 for every 500 HIV tests performed; and £200 for every new person diagnosed with HIV.

This work was designated as service evaluation and as such no ethical approval was required.

### Data sources

#### Laboratory surveillance (pre-study)

Data on HIV testing performed in Brighton and Hove (collected by the central laboratory) has been routinely audited since 2019 and published previously [13]. We provide additional data (available up until mid-2021) here.

#### Study data

The Doctors Laboratory provided summary data on the HIV tests performed as part of this study, with relevant clinical identifiers. Data on all blood test appointments during the study periods were extracted by each practice from their electronic health records. Four sites were able to provide data on each blood test appointment, including patient demographic variables and which staff member conducted the appointment. These two datasets were linked so that each blood test appointment was classified as not offered, offered but declined, tested, or ineligible (if offered within the last 12 months). Due to lack of engagement, three sites did not extract data.

After study completion, enrolled sites were sent an electronic questionnaire to be completed by staff that provided HIV testing (Supplementary Table S6). This was designed to assess their perspectives on the study, how they felt offering a HIV test, and identify any problems.

### Analysis

The characteristics of the blood test appointments were described using proportions or median/mean as appropriate. The prevalence of HIV-positivity was described as a percentage, with 95% confidence interval (CI).

Since patients may have multiple blood test appointments during the study period but can only be offered an HIV test once every 12 months, the following rules were applied. If a patient was offered an HIV test, that appointment was counted as their eligible appointment, regardless of whether it was their first appointment during the study period. They would then only become eligible again after 12 months. If a patient was never offered an HIV test during the study period, their first appointment was considered their eligible appointment.

To explore the association between patient's age, gender, the site, the staff, and incentive period with (i) the likelihood of being offered a test and (ii) the likelihood of a patient accepting a test, we used logistic regression modelling. To explore the impact of patient age and gender, we used mixed-effects logistic regression with site and staff as nested random effects. To explore the impact of staff and site, we opted to use them as fixed effects rather than random effects to allow for enumeration of odds ratios. Given collinearity between site and staff (individual staff were only present at one site) both could not be included as fixed effects in the same model. Odds ratios were calculated and presented with 95% CIs. To explore for linear associations, categorical variables were classified as ordinal variables in regression modelling. *P*-values were calculated through likelihood ratio testing comparing nested models.

To assess whether any association was consistent amongst sites, an interaction term was applied between the time period and site, with a *P*-value calculated by likelihood ratio test. If there was evidence of interaction, site-specific odds ratios and 95% CIs were calculated.

## Results

### Routine testing pre-study (2016–21)

Seven primary care facilities (Sites A–F) participated in this study. Between January 2016 and June 2021, data from routine laboratory surveillance reports that these sites conducted 3967 HIV tests, with a median of 7 tests per month (IQR: 4–12; Fig. 1). A total of 11 tests were positive, yielding an overall prevalence of 0.28% (95% CI: 0.14–0.50). The number of positive results per site ranged from 0 to 4 (Supplementary Table S1). Data on whether these cases represented new diagnoses were not available.

**Figure 1:**
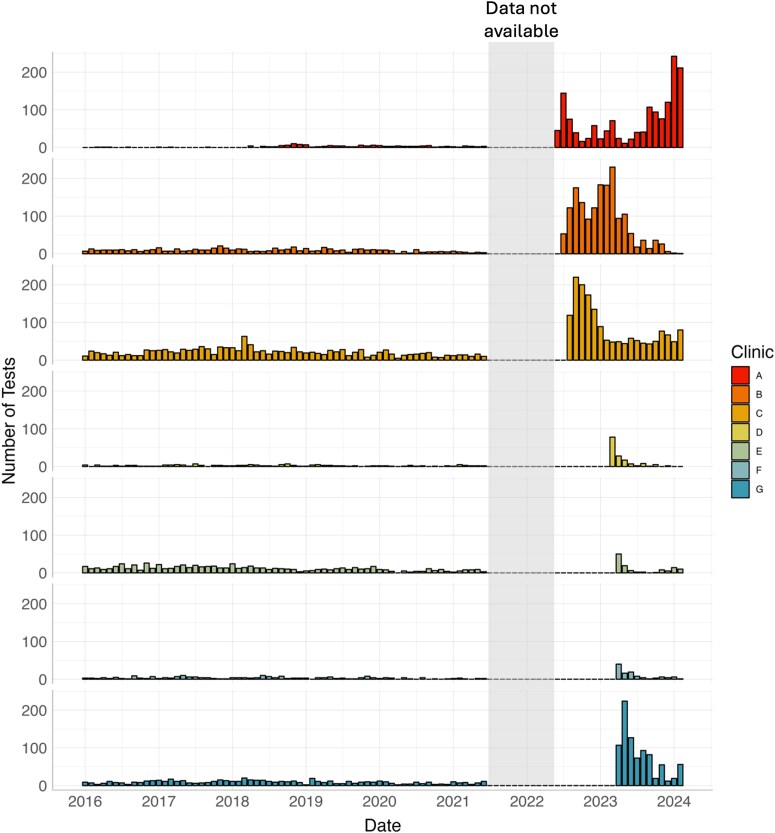
The number of tests (including repeat tests) monthly performed by the seven sites participating in the study. No data available at the time of publication for June 2021 to study start as audit had not been conducted on this data at time of writing. Note that only tests conducted as part of the study are included after 2022 (i.e. routine care samples are not included).

There was moderate evidence of an association between site and HIV prevalence amongst routinely performed tests (*P* = 0.04). Site A had the highest prevalence at 1.4% (95% CI: 0.2–5.3; Supplementary Table S1).

### Study implementation (2022–4)

Sites A–C joined the study in June 2022, while Sites D–G started in February 2023. Testing concluded in February 2024. Figure 1 illustrates the monthly number of tests performed before and during the study. Sites A–C conducted more than 1500 tests each, while Site G performed 867. Sites D–F each conducted fewer than 200 tests (Supplementary Table S2).

**Figure 2:**
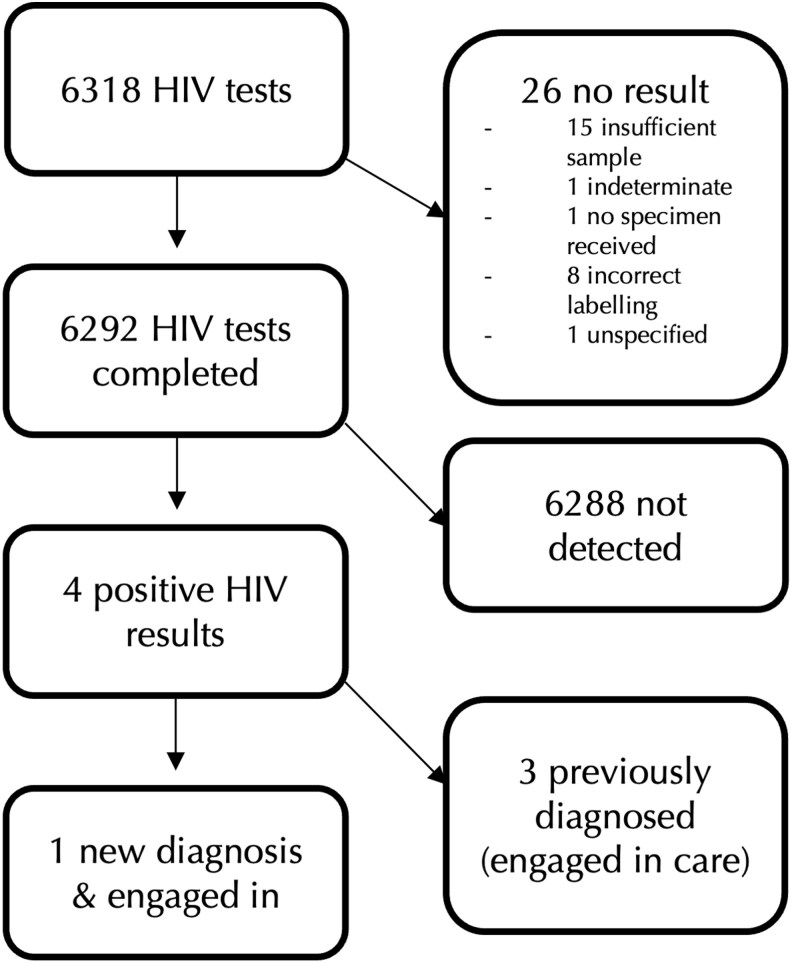
Diagram demonstrating the number of tests performed during the study, their results, and the outcomes.

In total, 6105 HIV tests were performed as part of the study. Of these, 4 were positive, 6075 were negative, and 26 (0.4%) were not processed (Fig. 2). Amongst the four positive results, three were from individuals known to be living with HIV and engaged in care, while one was a new diagnosis. This individual was successfully linked to care. The overall test positivity during the study was 0.07% (95% CI: 0.02–0.17), lower than the 0.28% (95% CI: 0.14–0.50) observed in routine testing from 2016 to 2021. When considering only new diagnoses, the incidence risk in the study was 0.016% (95% CI: 0.0004–0.09), equating to a number needed to test of 6250 (95% CI: 1091–240 384) to find one new person living with HIV.

### Offer and acceptance of HIV testing

We obtained data on all blood test appointments during the study period at Sites A–C and G.

Across the 4 sites, 33 433 blood test appointments were conducted, with 13 878 unique patients undergoing testing. The median number of blood test appointments per patient was 2 (IQR: 1–3), with 5558 patients attending only once (Supplementary Table S3). The study population was 57% female, with a median age of 58 (IQR: 33–68; Supplementary Table S4). Nine hundred and thirty-two (6%) were aged 81 years or older. Eighty-eight members of staff conducted blood test appointments during the study period performing a median of six HIV tests each (IQR: 1–216, mean 167, max. 1944; Supplementary Figure S1).

**Figure 3:**
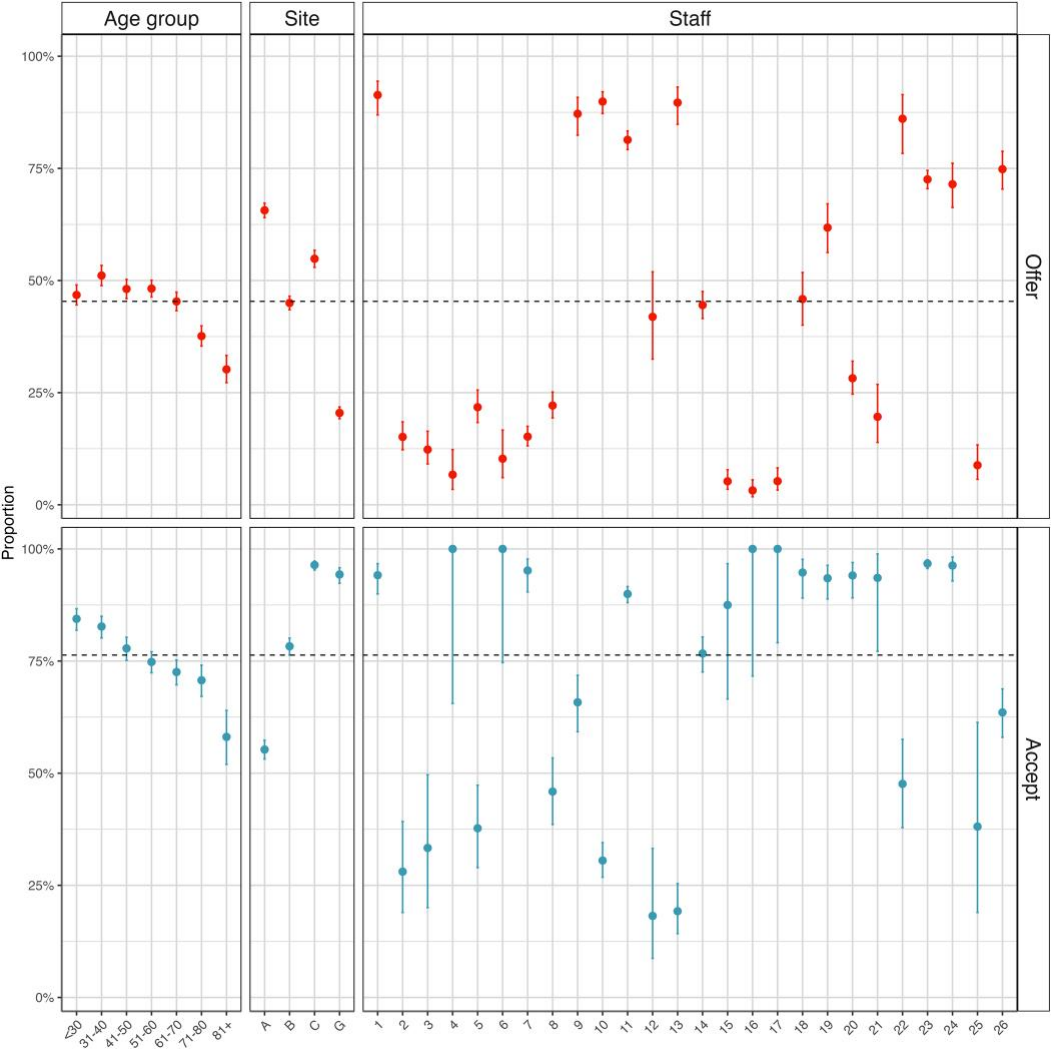
Proportions of patients offered and accepting a HIV test, by age, site, and staff member. Dashed line indicates overall proportion offered/accepted respectively. Error bars indicate 95% CIs. Only staff that conducted more than 100 blood test appointments during study period are displayed.

As patients became re-eligible for HIV testing 12 months after their last offer (whether accepted or declined), 14 649 patients were eligible for testing. A total of 6597 patients (45.0%) were offered HIV testing, and 4891 (74.1%) accepted (Fig. 3). Overall, 33.4% of eligible appointments resulted in HIV testing.

### Factors associated with testing offer and acceptance

The site, patient age, month, and attending staff member were strongly associated with both being offered a test and accepting a test (all *P* < 0.0001; Table 1, Supplementary Table S5). Patient gender was not associated with either outcome (*P* = 0.8 for offer, *P* = 0.3 for acceptance in multivariate analysis; Table 1).

**Table 1:** Association between being offered or accepting a HIV test and site, patient age, and patient gender.

		No. of patients	Number of tests offered (%)	Adjusted odds ratio (95% CI)	*P*-value	Number of tests accepted (%)	Adjusted odds ratio (95% CI)	*P*-value
Site^a^	A	3500	2322 (66.3)	Ref		1300 (56.0)	Ref	
	B	4434	2065 (46.6)	0.44 (0.40–0.48)		1476 (71.5)	2.70 (2.30–3.10)	
	C	3027	1454 (48.0)	0.47 (0.42–0.52)		1402 (96.4)	26.1 (19.4–35.9)	
	G	3688	756 (20.5)	0.13 (0.12–0.15)	<0.0001	713 (94.3)	15.0 (10.7–21.5)	<0.0001
Age	18–30	2059	939 (45.6)	Ref		786 (83.7)	Ref	
	31–40	2046	1020 (49.9)	1.09 (0.95–1.24)		826 (81.0)	1.07 (0.83–1.40)	
	41–50	2282	1065 (46.7)	1.01 (0.89–1.15)		820 (77.0)	0.84 (0.65–1.08)	
	51–60	2982	1433 (48.1)	1.13 (1.00–1.28)		1039 (72.5)	0.72 (0.57–0.91)	
	61–70	2411	1104 (45.8)	1.06 (0.93–1.20)		769 (69.7)	0.70 (0.55–0.89)	
	71–80	1920	744 (38.8)	0.79 (0.69–0.91)		489 (65.7)	0.62 (0.47–0.80)	
	81+	932	285 (30.6)	0.50 (0.42–0.60)	<0.0001	162 (56.8)	0.44 (0.32–0.61)	<0.0001
Gender	Female	8344	3764 (45.1)	Ref		2806 (74.5)	Ref	
	Male	6276	2820 (44.9)	1.0 (0.9–1.1)	0.8	2072 (73.5)	1.0 (0.9–1.2)	0.3

Odds ratio calculated through logistic regression adjusting for site (random effect), age, gender, month, and staff.

^a^For calculation of adjusted odds ratio for site, it was included as a fixed effect without staff in modelling. *P*-value calculated through likelihood ratio test of nested models. Data for staff was not presented here (*n* = 88).

The odds of being offered a test were similar for individuals aged 18–70 (95% CIs crossed 1, using 18–30 years as the reference). However, patients aged 71–80 and those over 80 were significantly less likely to be offered a test [aOR: 0.79 (95% CI: 0.69–0.91) and aOR: 0.50 (95% CI: 0.42–0.60), respectively]. Amongst those offered testing, adults over 50 were less likely to accept a test than younger individuals [aOR: 0.72 (95% CI: 0.57–0.91)], with the oldest age group (81+) least likely to accept [aOR: 0.44 (95% CI: 0.32–0.61)]. Figure 3 displays the proportion of patients offered and accepting a test by age.

Sites B, C, and G were less likely to offer HIV testing than Site A (*P* < 0.0001), with Site A offering testing to 66% of patients compared to 21% at Site G (Table 1). However, once offered a test, acceptance was higher at Sites B, C, and G, with Site C having a 96% acceptance rate compared to 56% at Site A (*P* < 0.0001, multivariate analysis; Table 1).

**Figure 4:**
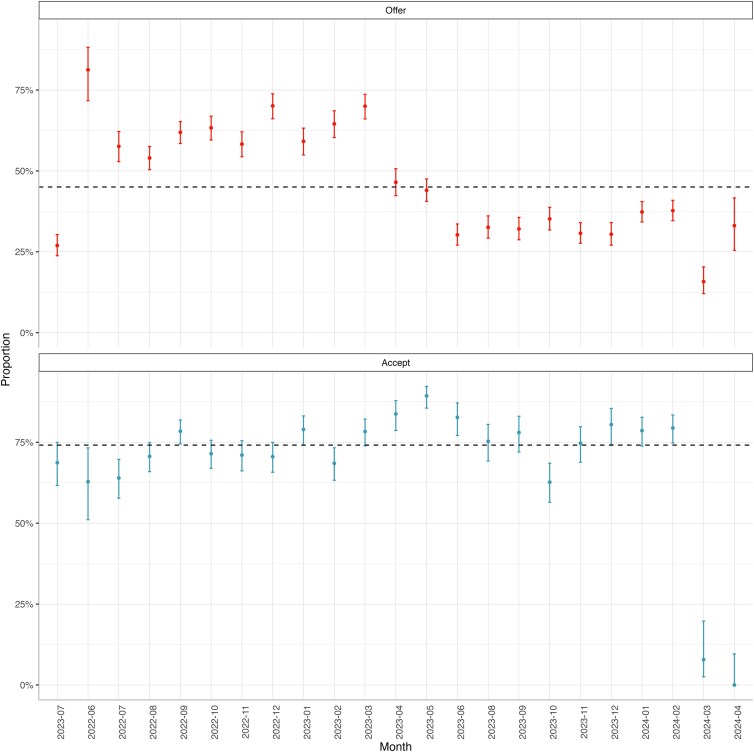
Proportion of patients being offered and accepting a HIV test by consultation month. Error bars indicate 95% CIs.

There was strong evidence that the staff member influenced both the likelihood of offering (*P* < 0.0001; Fig. 4) and accepting a test (*P* < 0.0001; Fig. 4). However, there was no evidence of correlation between a staff member's offer rate and patient acceptance (Spearman *P* = 0.1; Supplementary Figure S2).

We found strong evidence that both the likelihood of being offered a test and acceptance varied by month (*P* < 0.0001 for both; Fig. 4). Moreover, there was a clear linear decline in offer rates over time, with each additional month associated with a 4% decrease in the odds of being offered testing [aOR: 0.96 (95% CI: 0.95–0.96), *P* < 0.0001 for general vs. linear association; Fig. 4].

### Impact of financial incentives

From February 2023, financial incentives were introduced (details in Methods). Restricting analysis to Sites A–C (which were operational before incentives), we found that the proportion of patients offered a test declined from 61% pre-incentive to 47% during the incentive period [aOR: 0.54 (95% CI: 0.50–0.59), *P* < 0.0001]. There was weak evidence of a modest increase in acceptance rates during this period [aOR: 1.12 (95% CI: 0.99–1.27), *P* = 0.07].

The effect of financial incentives on offer rates varied significantly by site (*P* for interaction < 0.0001; Table 2). At Site A, the likelihood of offering a test remained stable [aOR: 1.11 (95% CI: 0.97–1.28)], whereas at Site C, the likelihood of offering a test fell sharply during the incentive period [aOR: 0.12 (95% CI: 0.10–0.14); Table 2].

**Table 2:** Multivariate association between offer and acceptance rates, and financial incentives.

		Offered							Accepted						
	No. of patients	No. of tests offered	Overall aOR (95% CI)	*P*-value	Site A-specific aOR (95% CI)	Site B-specific aOR (95% CI)	Site C-specific aOR (95% CI)	*P*-value (int.)	No. of tests accepted	Overall aOR (95% CI)	*P*-value	Site A-specific aOR (95% CI)	Site B-specific aOR (95% CI)	Site C-specific aOR (95% CI)	*P*-value (int.)
Pre-incentive	4922	2999 (60.9)	Ref		Ref	Ref	Ref		2176 (72.6)	Ref	0.07	Ref	Ref	Ref	
Incentive	6039	2842 (47.1)	0.54 (0.50–0.59)	<0.0001	1.11 (0.97–1.28)	0.80 (0.71–0.90)	0.12 (0.10–0.14	<0.0001	2002 (70.4)	1.12 (0.99–1.27)	0.07	3.29 (2.77–3.91)	0.28 (0.23–0.34)	0.62 (0.36–1.09)	<0.0001

Only Sites A–C included. Incentive defined as pre-incentive (pre-February 2023) and incentive (post-February 2023). Adjusted odds ratio calculated through mixed-effects logistic regression with site and staff as nested random effects, with patient age and gender as fixed effects. *P*-value calculated through likelihood ratio test of nested models. *P*-value for interaction [*P*-value (int.)] calculated through likelihood ratio test of nested models (interaction term between site and incentive period). Site-specific adjusted odds ratios calculated from regression model as previously described.

Similarly, the effect of incentives on acceptance rates varied by site (*P* for interaction < 0.0001; Table 2). At Site A, patients were more likely to accept testing during the incentive period [aOR: 3.3 (95% CI: 2.8–3.9)], whereas at Site C, acceptance declined [aOR: 0.28 (95% CI: 0.23–0.34); Table 2].

### Staff feedback

Sixteen staff members across the seven sites provided feedback via questionnaires. Respondents included 12 nurses (75%), 2 phlebotomists (12.5%), and 2 healthcare assistants (12.5%). All had performed at least 10 HIV tests, and half had conducted more than 100.

When asked about the ease of offering HIV testing, the most common response was ‘Very Easy’ (41%). No respondents found it difficult. All reported feeling at least ‘Somewhat Prepared’, with 56% selecting ‘Very Prepared’. Similarly, 87.5% (14/16) reported that they had sufficient pre-programme training and information.

Regarding patient reactions, most staff reported that patients were ‘Sometimes’ surprised by the offer, with 25% stating ‘Rarely’ and 6% indicating ‘All the time’. Thirteen respondents provided responses to open-ended questions. The most commonly suggested improvement was to ‘pre-prepare’ patients or increase patient awareness, with several respondents noting the potential usefulness of posters to support this. Full responses, including open-ended comments, are in Supplementary Table S7.

## Conclusions

We report on the largest study implementing routine HIV testing for patients undergoing blood tests in primary care within a high HIV prevalence area, in accordance with the 2016 NICE guidelines. Out of 6079 tests, one new HIV diagnosis was made, with a number needed to test (NNT) of 6079 (95% CI: 1091–240 384). The prevalence amongst tested patients was 0.07% (95% CI: 0.02–0.17), compared to 0.28% (95% CI: 0.14–0.50) observed in primary care testing between 2016 and 2021. In 2022, opt-out HIV testing was introduced in Emergency Departments, with Brighton and Hove participating. As of March 2024, 20 new diagnoses were made from 82 277 tests, yielding an NNT of 4114 (95% CI: 2664–6734), comparable to our primary care study (UKHSA, unpublished, communication with GD).

As expected, HIV prevalence was lower amongst patients tested in our study than amongst those tested pre-study in primary care. This is likely due to routine HIV testing being performed selectively—such as in response to indicator conditions, patient requests, or based on risk stratification. However, our study's NNT was similar to that of opt-out testing in Emergency Departments, albeit with wider CIs. Importantly, the prevalence of undiagnosed HIV in our study was below the 0.1% cost-effectiveness threshold [11]. Our findings provide a new estimate of undiagnosed HIV prevalence in Brighton and Hove and offer valuable insights into the feasibility of universal HIV testing in primary care. These results may inform policymakers in other high-prevalence areas (>2 per 1000 people aged 15–53).

This study builds on our earlier mixed methods review of a 3-week pilot at a single primary care centre, which demonstrated the feasibility of offering HIV testing as part of routine blood test appointments [12]. Amongst sites with complete data, 45% of eligible patients were offered an HIV test, similar to the 46% observed in our initial pilot at Site A [12]. This was higher than the 25% offer rate in the RHIVA2 trial of testing at 40 London general practices but lower than the 68% reported nationally for Emergency Department testing [10].

Unfortunately, three out of seven recruited sites withdrew from the study, effectively stopped providing study HIV tests, and did not provide blood test appointment data. The reasons for withdrawal are not known, but it is noteworthy that all three of these sites were recruited in the second round of recruitment potentially suggesting systematic differences in the on-boarding and training processes. The research team were available to support site teams as required, although regular meetings were not scheduled after the initial on-boarding process. Closer monitoring and further training (especially given the high levels of staff turnover and the importance of individual staff on offer and acceptance rates) may have mitigated.

Although the study was successfully implemented at the remaining four sites, based on high testing numbers, barriers remained to maximizing the proportion of patients offered a test. In our post-survey, all respondents rated offering a HIV test as ‘neutral’, ‘easy’, or ‘very easy’, with most feeling ‘very prepared’ to offer testing. However, despite that, test offers declined month by month, suggesting waning engagement.

To address lower-than-expected testing numbers, financial incentives were introduced to encourage participation. However, these incentives did not prevent the decline in test offers overall. At Site A, offer rates remained stable, whereas they declined at Sites B and C. Our findings indicate that individual staff play a crucial role in such programmes, with staff turnover having significant consequences. We hypothesize that sustained engagement, recognition of high-performing staff, and real-time audit and quality improvement efforts could enhance offer rates.

We also found that test offer rates varied by patient age, with those over 70 years being less likely to be offered an HIV test. This is a critical finding, as older adults in the UK have the highest rates of late HIV diagnosis (CD4 < 350 at diagnosis), which is associated with increased morbidity, mortality, and healthcare costs [14]. Ensuring that universal HIV testing programmes offer tests indiscriminately is essential.

Building on the universally positive feedback from our earlier pilot, 74% of patients in this study accepted an HIV test when offered, compared to 69% in the RHIVA2 trial and 79% in our initial pilot [10, 12]. As with test offers, acceptance rates were lower amongst older adults. Additionally, acceptance varied by site, with lower rates at Site A, and by staff suggesting differences in how test offers were presented.

There are several limitations to our study. First, three of the seven sites withdrew from the study and we were unable to extract data on their blood test appointments. We were unable to explore the reasons for lack of engagement or identify potential solutions. Overall, this likely led to an overestimation of the proportion of eligible patients who were offered and accepted testing. Nevertheless, our findings demonstrate that with appropriate support, routine HIV testing in primary care can be implemented and is acceptable to patients. Second, sites were supported in documenting whether a test was offered, but as with all routinely collected clinical data, misclassification remains a possibility. Finally, while we sought to understand barriers to site engagement via questionnaires, these were likely completed by staff already engaged in the study, potentially overestimating acceptability.

Our study demonstrates that offering HIV testing to patients undergoing routine blood tests in primary care is acceptable to patients and can be integrated into routine clinical practice. However, further research is needed to evaluate the feasibility and long-term sustainability of this approach, as three sites withdrew from the study and testing uptake declined over time. In addition, with only one new diagnosis identified amongst 6000 tests, formal evaluation of cost-effectiveness is required before wider implementation. The effectiveness of this strategy is likely to depend on local context and underlying prevalence of undiagnosed HIV. Nevertheless, routine universal-offer testing may represent a valuable component of efforts to reduce HIV transmission in the UK, particularly if a model of presumed consent—whereby testing is undertaken unless actively declined—is adopted.

## Supplementary Material

cmag038_Supplementary_Data

## Data Availability

Anonymized data can be made available for scientific purposes upon reasonable request to the corresponding author.
