# Supplementary material for: Universal-offer HIV testing amongst patients undergoing blood tests in primary care: results from a multi-centre study
Source: Fam Pract. 2026 Jun 30;43(4):cmag038. doi: 10.1093/fampra/cmag038 (PMC13320703; doi:10.1093/fampra/cmag038)

Supplementary Materials

Site	No. of tests performed	No. of positive tests	Prevalence, % (95% CI)	P-value
A	147	2	1.4 (0.2-5.3)	
B	606	4	0.7 (0.2-1.8)	
C	1371	2	0.1 (0.0-0.6)	
D	280	2	0.7 (0.1-2.8)	
E	745	1	0.1 (0.0-0.9)	
F	219	0	0.0 (0.0-2.1)	
G	599	0	0.0 (0.0-0.8)	0.04

Supplementary Table 1 – By site, number of tests, number of positive tests and the test prevalence between 2016 and 2021 (pre-Study). P-value calculated from likelihood ratio test of nested logistic regression models.

Site	No. of HIV tests (%)	Number of weeks	Median No. of weekly tests (IQR)
A	1527 (25.0)	88	25 (15 – 46)
B	1687 (27.6)	83	37 (18 – 48)
C	1651 (27.0)	80	25 (13 – 49)
D	148 (2.4)	52	3 (2 – 6)
E	117 (1.9)	46	3 (1 – 5)
F	108 (1.8)	46	2 (1 – 4)
G	867 (14.2)	45	14 (9 – 25)

Supplementary Table 2 – Number of tests conducted by site during the study, number of weeks operating, and the median number of weekly tests conducted by site. % represents the proportion of all tests conducted (n=6105).

Number of Blood Test Appointments	Number of patients
1	5558
2	3238
3	1762
4	1108
5	689
6	429
7	241
8	154
9	130
10+	117

Supplementary Table 3 – Frequency distribution of the number of blood test appointments attended by patients at sites A, B, C, and G.

Group	Number of patients (%)
**Age**	
18 – 30	2059 (14.0)
31 – 40	2046 (14.0)
41-50	2282 (15.6)
51-60	2982 (20.4)
61-70	2411 (16.5)
71-80	1920 (13.1)
81+	932 (6.4)
Missing	17 (0.1)
**Gender**	
Female	8344 (57.0)
Male	6276 (42.8)
Missing	29 (0.2)

Supplementary Table 4 – Frequency distribution of the patient demographics at eligible blood test appointments (n=14649). Age in years.

		No. of patient s	Number of tests offered (%)	Odds Ratio (95% CI)	P-value	Number of tests accepted (%)	Odds Ratio (95% CI)	P-value
**Site***	A	3500	2322 (66.3)	ref		1300 (56.0)	ref	
	B	4434	2065 (46.6)	0.44 (0.40-0.48)		1476 (71.5)	2.70 (2.30-3.10)	
	C	3027	1454 (48.0)	0.47 (0.42-0.52)		1402 (96.4)	26.1 (19.4-35.9)	
	G	3688	756 (20.5)	0.13 (0.12-0.15)	<0.0001	713 (94.3)	15.0 (10.7-21.5)	<0.0001
**Age**	18 – 30	2059	939 (45.6)	ref		786 (83.7)	ref	
	31 – 40	2046	1020 (49.9)	1.19 (1.05-1.34)		826 (81.0)	1.07 (0.83-1.40)	
	41-50	2282	1065 (46.7)	1.04 (0.93-1.18)		820 (77.0)	0.84 (0.65-1.08)	
	51-60	2982	1433 (48.1)	1.10 (0.99-1.24)		1039 (72.5)	0.72 (0.57-0.91)	
	61-70	2411	1104 (45.8)	1.01 (0.90-1.13)		769 (69.7)	0.70 (0.55-0.89)	
	71-80	1920	744 (38.8)	0.75 (0.66-0.86)		489 (65.7)	0.62 (0.47-0.80)	
	81+	932	285 (30.6)	0.53 (0.45-0.62)	<0.0001	162 (56.8)	0.44 (0.32-0.61)	<0.0001
**Gender**	Female	8344	3764 (45.1)	ref		2806 (74.5)	ref	
	Male	6276	2820 (44.9)	1.0 (0.9-1.1)	0.8	2072 (73.5)	0.9 (0.8-1.1)	0.3

Supplementary Table 5 – Univariate association between being offered or accepting a HIV test, and Site, patient age, and patient gender. Odds Ratio calculated through univariate logistic regression. P-value calculated through likelihood ratio test of nested models. Data for staff was not presented here (n=88)

Electronic survey questions

1. Which practice do you work at?
2.What is your role?
3.How easy did you find it to offer HIV testing? Very Difficult, Difficult, Neutral, Easy, Very Easy
4.How prepared were you to offer HIV testing? Not at all, Not very, somewhat, Well, Very Prepared
5.Do you feel you had sufficient pre-programme training and information? Yes, No, Not sure
6.How often were patients surprised by the offer of a test? Never, Rarely, Sometimes, Often, All the time
7.How many patients did you test for HIV during the entire testing period? (roughly), <10, 10-99, >100
8.How would you improve the HIV testing programme if offered elsewhere? (free text)
9.Please provide any other comments on the HIV testing programme. (free text)

How would you improve the HIV testing programme if offered elsewhere?
a standard text to patients booking for bloods that they will be offered this
I think the posters were very helpful in preparing people for the question. Maybe a poster aimed at >65s would help as they were group most likely to think test unnecessary.
I feel more leaflets/posters were handed and on display once the programme was finished. If next time we were to do the programme, I feel information should be sent to patients who consent to text messages to advise we would be doing HVI testing and to request during a blood test if they would like to, posters on display and leaflets to take on display
Uniform approach to pre preparing patients that it would be offered prior to the appointment - I feel this would increase uptake
na - all was well, good information, good processes.
Awareness
Can't think of any way to improve the programme, but we did find a lot of repeat patients coming back for bloods in the time period [ie. frequent attenders at the surgery], so became more unlikely we were able to offer to untested individuals within the testing period.
may be include it as part of routine annual blood tests., would you still need pt consent for this?
Maybe a little more awareness - most patients absolutely fine but some were 'surprised' and a little taken aback by the test and declined for this reason.
It was very straightforward im not sure it could be simplified
Not have to add an extra bottle- I know if goes to a different department, but if having lots of bloods taken, sometimes patients were not keen on having the extra bottle and also if difficult to bleed, it was left off for those patients etc.
do it more!

Supplementary Table 6 – Open-ended responses to questionnaire (“How would you improve the HIV testing programme if offered elsewhere?

Please provide any other comments on the HIV testing programme.
Only negative was that it wasn't obvious who we had offered it to and/taken a sample for already without going into the template first.
Big effort tbh but worth it.
Brilliant programme for ensuring blanket testing without predjudice
Thank you for all you do!
As above, so inconvenient for courier and presumably high costs for low yield.
pts sometimes get offended, maybe iif it was done annuallly it might remove the stigma associated?

Supplementary Table 7 – Open-ended responses to questionnaire ("Please provide any other comments on the HIV testing programme")

Example of financial incentive system:

1. 400 tests performed and no positives -> £800
2. 600 tests performed and no positives -> £1400 (£1200 tests, £200 500 test bonus)
3. 600 tests performed and one positive -> £1600 (£1200 test, £200 500 test bonus and £200 positive bonus

Supplementary Figure 1 – Distribution of the number of blood test appointments by staff member during study period.

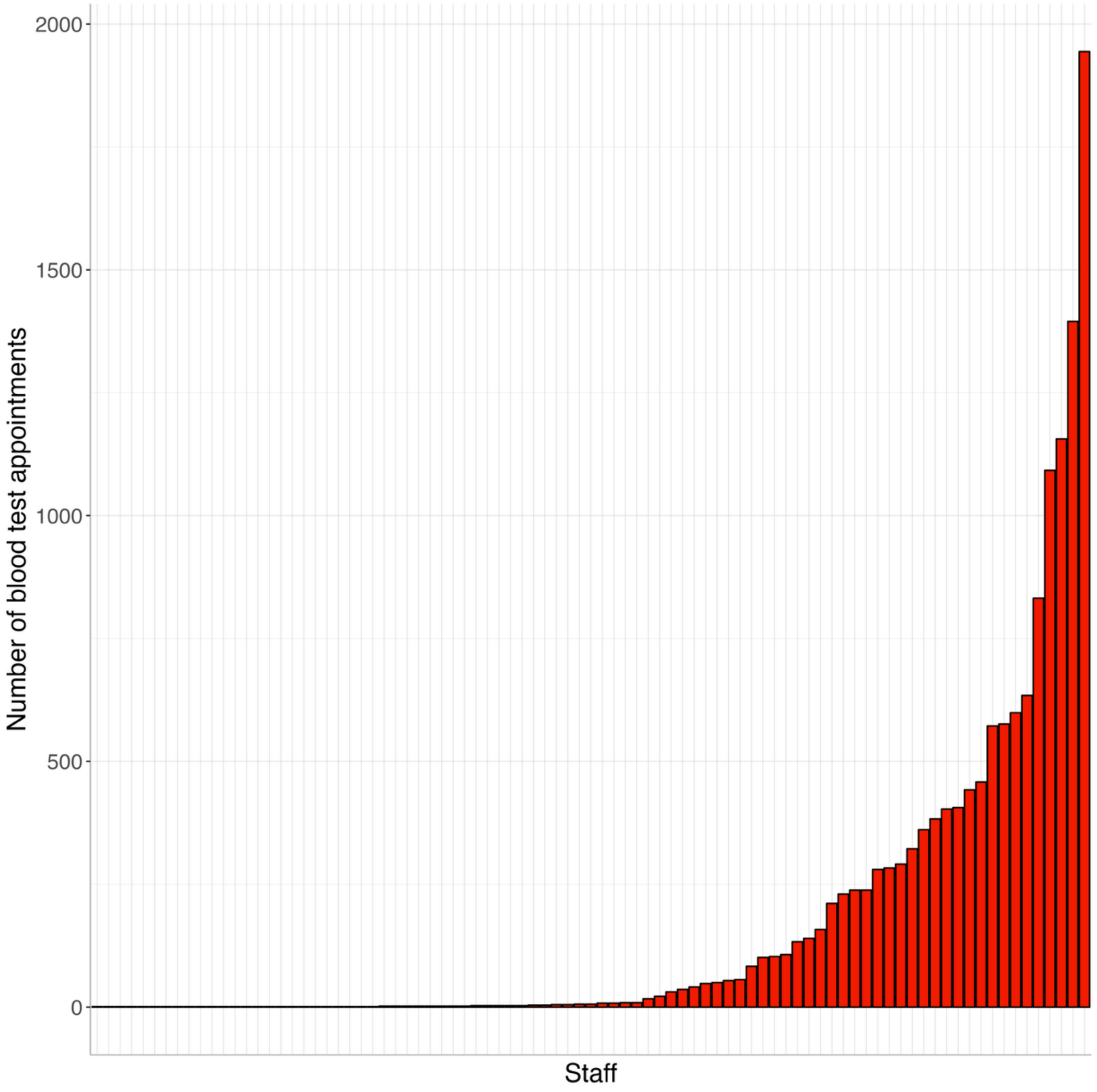

Supplementary Figure 2 – Association between the offer and acceptance rate per staff. Regression line as blue line, with 95% confidence intervals as shaded area.

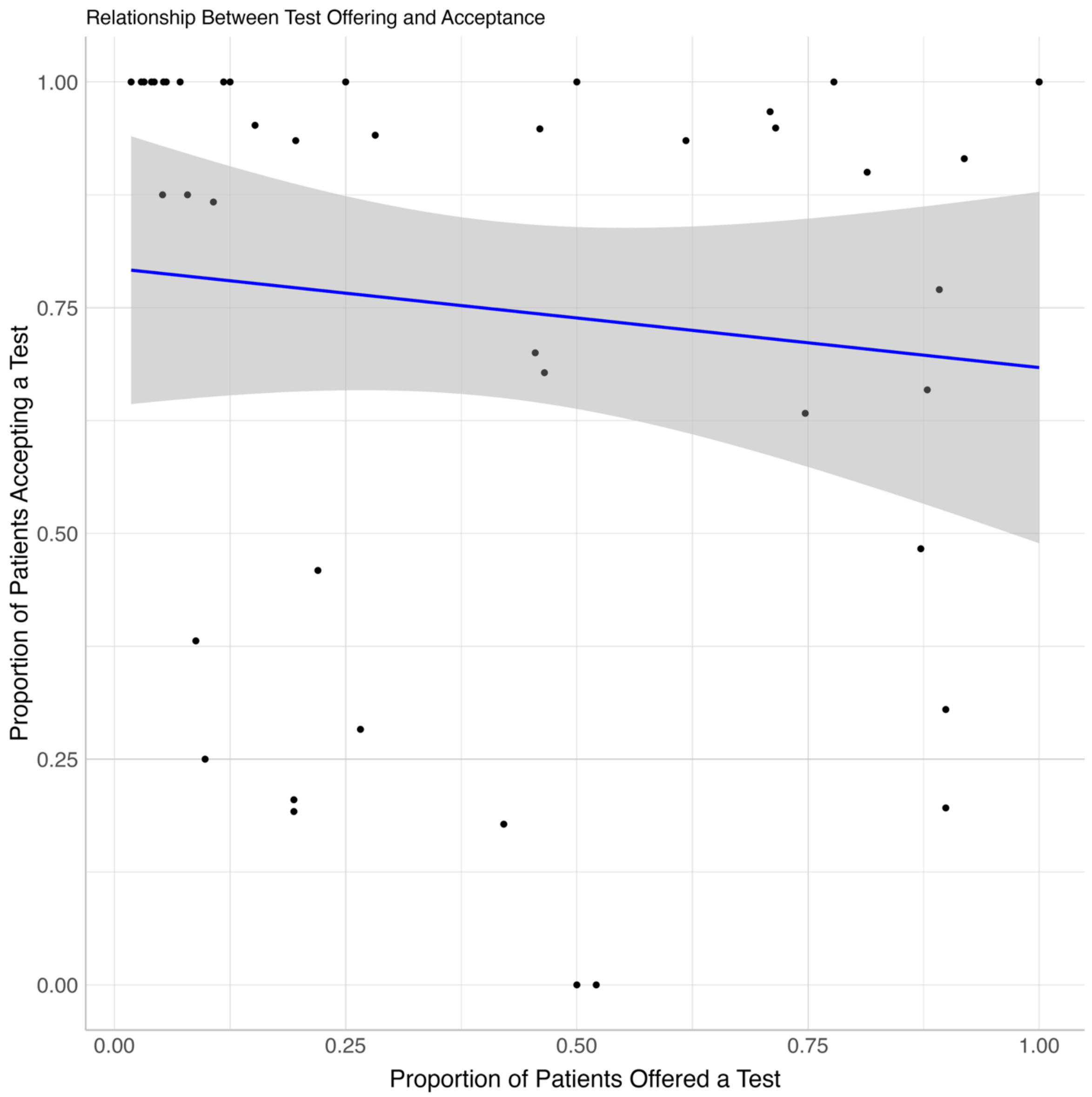

Supplement: cmag038_Supplementary_Data [file cmag038_supplementary_data.pdf]
